# An outbreak of Shigella sonnei infection associated with consumption of iceberg lettuce.

**DOI:** 10.3201/eid0101.950105

**Published:** 1995

**Authors:** J A Frost, M B McEvoy, C A Bentley, Y Andersson

**Affiliations:** Laboratory of Enteric Pathogens, Central Public Health Laboratory, London, England, UK.


9. Merianos A, Farland AM, Patel M, Currie B, Whelan P, Dentith H, Smith D. A concurrent outbreak of Barmah Forest and Ross River disease in Nhulunbuy, Northern Territory. Comm Dis Intell (Aust) 1992;16:110-1.
10. Broom AK, Mackenzie JS, Lindsay MD, Wright AE. Epidemiology of Murray Valley encephalitis and Kunjin viruses in Western Australia, 1980-89. Arbovirus Research in Australia 1989;5:14-8. CSIRO and Queensland Institute of Medical Research.
11. Lindsay MD, Johansen C, Wright AE, Condon R, D'Ercole M, Smith D, Mackenzie JS. The epidemiology of outbreaks of Ross River virus infection in Western Australia in 1991-1992. Arbovirus Research in Australia 1992;6:72-6. CSIRO and Queensland Institute of Medical Research.
12. Lindsay MD, Broom AK, Wright AE, Johansen CA, Mackenzie JS. Ross River virus isolations from mosquitoes in arid regions of Western Australia: implication of vertical transmission as a means of persistence of the virus. Am J Trop Med Hyg 1993;49:686-96.

### An Outbreak of *Shigella sonnei* Infection Associated with Consumption of Iceberg Lettuce

**To the Editor:** *Shigella sonnei* outbreaks in England and Wales are typically associated with primary schools and nurseries. The mode of transmission is usually from person to person by the fecal-oral route (1). In a June 1994 outbreak of *Sh. sonnei* food poisoning among adults in several countries in North West Europe, the vehicle of infection appeared to be iceberg lettuce (2).

In early June, the Communicable Disease Surveillance Centre (CDSC), Public Health Laboratory Service, received a report of an increase in domestic cases of *Sh. sonnei* infections in Sweden from the Salmnet network—a European international laboratory-based reporting system for human salmonella infections that provides a timely on-line database. In this instance the network was used for shigellosis. Of 100 reported cases of *Sh. sonnei* infection in Sweden, 52 occurred in two outbreaks in mid-May. Many cases seemed to be due to foodborne infection, and iceberg lettuce and peeled frozen prawns were implicated as vehicles of infection. *Sh. sonnei* phage types 2 and 3 alpha were associated with the outbreaks, and phage types 2 and 65 had been isolated from sporadic cases.

A message was sent throughout England and Wales on Epinet (a system for rapid electronic data transfer to all Consultants in Communicable Disease Control [CsCDC] in each District Health Authority, Public Health Laboratories [PHLs] and other agencies involved in infectious disease control) asking for information on possible foodborne *Sh. sonnei* infection to be sent to CDSC and for isolates to be referred to the Laboratory of Enteric Pathogens (LEP) for phage typing.

### Epidemiologic studies

Laboratory reports of *Sh. sonnei* infection received through the routine reporting system at CDSC were scrutinized to determine the age group and sex distributions during weeks 21 to 24.

After the Epinet message, CsCDC and laboratory directors who reported clinical cases for which *Sh. sonnei* was isolated were asked to administer trawling questionnaires to apparently sporadic cases among adults with no recent history of overseas travel. Personal details and history of illness and exposure to particular foods were sought.

Several small outbreaks and clusters were reported during June. CsCDC was asked for results of any analytical epidemiologic studies to CDSC. The results of the national laboratory reporting system are shown in Table 1. Although there were fewer reports in the first 20 weeks of 1994 than in a similar period in 1993, there were more reports in the weeks 21 to 24 and many more reports among adults. The proportion of total reports constituted by those from adults was 66% in weeks 21 to 24 of 1994 compared with 44% with the same period in 1993. The proportion in women in the 2 periods was 42% in 1994 compared with 26% in 1993.

Forty trawling questionnaires were distributed. Almost all case patients (38/40) had eaten various salad items of which the common food was iceberg lettuce. The lettuce had been consumed in restaurants, pubs, and in the homes of the case-patients. The lettuce was purchased from supermarkets, greengrocers' shops, and street markets. In one outbreak in Northampton, 21 (52%) of guests at a party became ill with diarrhea. *Sh. sonnei* was isolated from fecal specimens. Illness was significantly associated with consumption of iceberg lettuce (relative risk 3.68, confidence intervals 1.34 - 10.11, $p = 0.0004$).

The hypothesis that consumption of iceberg lettuce was associated with apparently sporadic *Sh. sonnei* infection in adults was tested by a case-con-

**Table 1. *Shigella sonnei* in England and Wales—Laboratory reports to CDSC**

| Year | Number of reports (%) | | | | |
|---|---|---|---|---|---|
| | Week 1–20 | Weeks 21–24 | | | Weeks 1–24 |
| | Total | Total | Adults | Women | Total |
| 1993 | 3190 | 480 (100) | 211 (44) | 127 (26) | 3670 |
| 1994 | 1557 | 505 (100) | 333 (66) | 214 (42) | 2062 |

trol study. A case was defined as a person aged 14 or more years who became ill after May 1, 1994, and had microbiologic evidence (fecal isolation) of *Sh. sonnei* infection, no recent history of overseas travel, and no identifiable contact with other case-patients in the 3 days before onset. Controls were nominated by case-patients and matched by sex, age (within a 10-year age band), and area of residence (within a 10-mile radius of the case). For each case three matched controls were sought. A questionnaire was administered by telephone by three interviewers from CDSC. Clinical and demographic details and details of exposure to food items, including iceberg lettuce, mentioned in trawling questionnaires were sought.

Twenty-eight case-patients and 49 matched controls were interviewed and, after excluding those who had recently traveled abroad and controls who had been ill, results from 27 cases and 44 controls were analyzed. The median age of case-patients was 47 years, and the range was 19 to 79 years. Eight cases were among men and 19 among women. All case-patients had diarrhea (i.e., three or more loose stools in a 24-hour period), although only four of the 27 reported blood in the stools, 25 of the 27 had abdominal pain, and 11 reported vomiting. The median duration of symptoms was 9 days, and the range was 4 to 25 days. Taking into account the matching inherent in the study design, a matched analysis was performed. In any analysis 27 matched sets were possible. For 13 sets there was one control per case, for 11 sets there were 2 per case, and for 3 sets there were 3 controls per case. Single variable analysis of the different foods consumed revealed the possible risk factors ($p < 0.2$) (Table 2). A multivariable model was fitted with all those variables included. This procedure was repeated, removing nonsignificant items at each stage. In the third model, the only remaining significant item was iceberg lettuce ($p = 0.0172$). The estimated odds ratio for iceberg lettuce was 13.8 (95% confidence interval 1.26 to 150.5).

In sporadic cases associated with consumption of lettuce from particular restaurants or public houses, it was possible to compare the date of onset with the date of delivery of iceberg lettuce by the wholesalers. The distribution chain was traced back through importers supplying wholesale markets in England. The wholesalers were supplied by packers in Spain. This was consistent with the findings of the investigators in the Norwegian outbreak. Iceberg lettuce investigated by the Public Health Laboratory service during the second week of June 1994 did not grow *Sh. sonnei*. However, the iceberg lettuce season in Spain, which began in October, ended early in June, and the source of lettuce available for testing could not be traced.

### Laboratory studies

All *Sh. sonnei* isolates referred to LEP after the Epinet message were phage typed by using the scheme described by Hammerstrom (3) and Kallings and Sjoberg (4), according to a protocol supplied by Dr. R. Wollin, Swedish Institute of Infectious Disease Control, Sweden. Isolates were also tested for resistance to a range of antibiotics by an agar dilution technique (5).

A total of 495 isolates were referred to LEP between June 14 and July 31, from 51 laboratories in England and Wales. Most isolates were from sporadic infections, but in a number of local outbreaks, there was a strong epidemiologic association between illness and consumption of iceberg lettuce. Two phage types predominated among the 19 types identified during this period, PT 2 (42.6% of isolates) and a variant of PT65 provisionally designated PT L (15.9%). In contrast, although a small number of isolates of PT 65 and PT L were identified among strains of *Sh. sonnei* isolated in England and Wales in 1991 and 1992, no isolates of PT 2 were seen before May 1994. Towards the end of the outbreak PT 3 and PT 6 were becoming reestablished in England and Wales as the predominant types, as they had been in previous years.

An exception to the recent pattern was an outbreak in North Wales, involving several children and adults, in which infection was associated either with eating ice cream at a particular establishment or having contact with children who had done so. All 73 of the isolates of PT 62 were associated with this outbreak.

A total of 357 *Sh. sonnei* isolated during this period (72.1%) were fully sensitive to all drugs

**Table 2. *Sh. sonnei* case control study—Single variable matched analysis**

| | Case (n=27) | | | Control (n=44) | | | | |
|---|---|---|---|---|---|---|---|---|
| **Food** | **Ate** | **Did not eat** | **% ate** | **Ate** | **Did not eat** | **% ate** | **No. of sets** | **p-value** |
| Prawns | 4 | 22 | 15 | 7 | 37 | 15 | (26s) | 0.9388 |
| Shrimps | 0 | 27 | 0 | 1 | 43 | 2 | (27s) | 0.3070 |
| Steak | 7 | 19 | 27 | 6 | 37 | 14 | (26s) | 0.0139 |
| Burgers | 5 | 22 | 19 | 12 | 32 | 27 | (27s) | 0.2045 |
| Salad | 25 | 2 | 93 | 34 | 10 | 77 | (27s) | 0.0081 |
| Cold meats | 15 | 10 | 60 | 29 | 13 | 69 | (23s) | 0.7708 |
| Tomatoes | 22 | 4 | 85 | 33 | 11 | 75 | (26s) | 0.1039 |
| Spring onions | 5 | 21 | 19 | 9 | 35 | 21 | (26s) | 0.7556 |
| Celery | 5 | 22 | 19 | 5 | 39 | 11 | (27s) | 0.2956 |
| Cucumber | 16 | 11 | 59 | 31 | 13 | 71 | (27s) | 0.3798 |
| Other salads | 16 | 10 | 62 | 26 | 18 | 59 | (26s) | 1.0000 |
| Lettuce | 25 | 2 | 93 | 31 | 13 | 71 | (27s) | 0.0007 |
| Cos lettuce | 2 | 20 | 9 | 3 | 25 | 11 | (17s) | 1.0000 |
| Webb's lettuce | 5 | 18 | 22 | 4 | 34 | 11 | (21s) | 0.2632 |
| Lamb's lettuce | 2 | 17 | 11 | 6 | 36 | 14 | (19s) | 0.1002 |
| Raddicio lettuce | 4 | 20 | 17 | 7 | 34 | 17 | (23s) | 0.8720 |
| Iceberg lettuce | 17 | 8 | 68 | 19 | 23 | 45 | (25s) | 0.0023 |
| Frisee lettuce | 5 | 18 | 22 | 4 | 37 | 10 | (22s) | 0.2582 |
| Home | 15 | 8 | 65 | 27 | 16 | 63 | (23s) | 0.8494 |
| Restaurant | 6 | 16 | 27 | 5 | 33 | 13 | (21s) | 0.1546 |
| Pub | 4 | 18 | 18 | 2 | 34 | 6 | (20s) | 0.0795 |
| Other outlet | 10 | 14 | 42 | 7 | 31 | 18 | (22s) | 0.0012 |

tested. Phage types 2 (87% fully sensitive) and PT L (99%) were predominantly sensitive, as were all isolates of PT 62; usually one would expect more than 70% of *Sh. sonnei* isolates to be resistant to one or more drugs. The use of the same phage-typing scheme across several European countries has facilitated cross-referencing between the British, German, and Swedish outbreaks. Phage types 2 and 65 (or the closely related variant PT L) were identified in several countries.

From the epidemiologic studies, it was concluded that the strong statistical evidence ($p = 0.0172$) that consumption of iceberg lettuce was associated with the risk of becoming ill together with reports from other European countries, including Scotland, Sweden, and Norway, and the temporal association of the outbreak with the iceberg lettuce season in Spain implicated iceberg lettuce as the vehicle of infection. This was corroborated by the laboratory studies, which showed a change in predominant phage types during the period of the outbreak. The predominance of the same phage types in lettuce-associated *Sh. sonnei* infections in a number of countries added further weight to this conclusion.

In England, there were several anecdotal accounts of dual infection with salmonellae and viruses as well as *Sh. sonnei*. This was also true of infections in Norway and Sweden (6). A plausible explanation would be that fecally contaminated water was used to irrigate the lettuce or to cool it after packing. If iceberg lettuce is not washed thoroughly before consumption, contamination could be retained in the leaves.

This study demonstrates both the importance of coordinating laboratory results and epidemiologic investigations and the value of rapid communications and common typing techniques in various European countries.

**J.A. Frost, M.B. McEvoy,* C.A. Bentley, Y. Andersson,** and B. Rowe**
Laboratory of Enteric Pathogens,
Central Public Health Laboratory, London, England
*Communicable Disease Surveillance Centre, London, England
**Swedish Institute for Infectious Diseases Control, Stockholm, Sweden

### References

1. Newman CPS. Surveillance and control of *Shigella sonnei* infection. Communicable Disease Report 1993; 3: R63-8.
2. Anonymous. A foodborne outbreak of *Shigella sonnei* infection in Europe. Communicable Disease Report 1994; 4 No. 25.

3. Hammerstrom E. Phage typing of *Shigella sonnei*. Acta Med Scand 1949. 133 (Suppl 223).
4. Kallings LO, Lindberg AA, Sjoberg L. Phage typing of *Shigella sonnei*. Arch Immun Ther Exp 1968; 16: 280-7.
5. Frost JA. Testing for resistance to antimicrobial drugs. In: Chart H, ed. Methods in practical laboratory bacteriology. Boca Raton Fla.: CRC Press; 1994.
6. Kapperud G, Rorvik LM, Hasseltvedt V, et al. Outbreak of *Shigella sonnei* infection traced to imported iceberg lettuce. J Clin Microbiol, in press.

### ?Lyme Disease in Australia— Still To Be Proven!

**To the Editor:** The first case of a syndrome consistent with Lyme disease was reported from the Hunter Valley region of New South Wales (NSW) in southeastern Australia in 1982, but there was no confirming serology. More clinical cases, again without serologic confirmation, were reported in 1986, two from the south coast and one from the central coast of NSW. The Queensland State Health Laboratories reported that 186 (14.9%) of 1,247 sera taken from patients between 1986-1989 showed antibody response to *Borrelia burgdorferi* of ≥ 64 by indirect fluorescence antibody test (IFAT), but none of these results were confirmed by immunoblotting.

In 1988, a multidisciplinary investigation of putative Lyme disease began, encompassing clinical, serologic, vector, and reservoir host studies, and results from these studies have been published (1). What follows herein is derived from the accumulated published and unpublished data of the research team, the members of which are credited in the acknowledgments.

Over the past 6 years, principally because of local publicity, there has been an increase in serologic testing for Lyme disease in Australia, particularly in southeastern Australia. Testing has often been initiated by patients with undiagnosed health problems. Thus, most Lyme disease patients seen by infectious disease specialists are self selected and are referred for assessment on the basis of tick exposure and reported positive serologic test results for Lyme disease.

Patients with positive serologic test results frequently have long-standing symptoms for which no other diagnosis has been established. The most common symptoms are musculoskeletal, including myalgias and arthralgias without objective evidence of joint swelling, and syndromes involving fatigue and loss of energy resembling chronic fatigue syndrome. Some patients fulfill diagnostic criteria for fibromyalgia. The next most common symptoms are neurological, and include frequent headaches, inability to concentrate, and memory loss. The most common dermatologic manifestation of chronic Lyme disease, acrodermatitis chronica atrophicans, seen occasionally in Europe and rarely in the United States, has not been reported from Australia.

A few cases of *erythema migrans*, the characteristic dermatologic manifestation of acute Lyme disease, have been reported from southeastern Australia, but clinical diagnosis can be confounded by hypersensitivity reactions to tick bite; a spectacular erythematous reaction is often associated with the bite of *Ixodes holocyclus*, the most common tick biting humans in NSW. Only eight specimens submitted to our laboratory included skin biopsies done to isolate spirochetes. *B. burgdorferi* s.l. was isolated from one patient returning from Europe, but no spirochetes were isolated from local patients.

In our serologic diagnostic service, an enzyme-linked immunosorbent assay (ELISA) for IgG and an IFAT for IgG and IgM have been used with antigens derived from North American *B. burgdorferi* strain B31 (2). From 1988 to April 1994, 78 (1.8%) of 4,372 local patients were positive for IgG by both methods. All 78 patients were tested by IgG Western blot for confirmation by using the virulent North American *B. burgdorferi* strain 297 and a German strain designated B7: with *B. burgdorferi* strain 297, 46 patient samples showed as many as four indicative bands; with the European strain B7, 22 patient samples showed as many as three indicative bands; bands used were 18, 21, 28, 30, 31, 34, 39, 41, 45, 58, 66, 83, and 93 kDa, modified from Dressler et al (3). Twenty-four other patients with various bacterial, viral, or autoimmune syndromes not relating to Lyme disease were tested as controls: with strain 297, 11 control samples showed as many as two indicative bands, and with strain B7, 10 control samples showed as many as two indicative bands.

A high degree of cross-reactivity was demonstrated with the controls, particularly with respect to the 31, 41, 58, and 66 kDa bands for both the European and the American antigen. As none of the 78 patients, including putative late-stage patients positive by ELISA and IFAT, showed more than four
